# Effects of Low Protein Diet on Modulating Gut Microbiota in Patients with Chronic Kidney Disease: A Systematic Review and Meta-analysis of International Studies

**DOI:** 10.7150/ijms.66451

**Published:** 2021-10-25

**Authors:** Cheng-Kai Hsu, Shih-Chi Su, Lun-Ching Chang, Shih-Chieh Shao, Kai-Jie Yang, Chun-Yu Chen, Yih-Ting Chen, I-Wen Wu

**Affiliations:** 1 Department of Nephrology, Chang Gung Memorial Hospital, Keelung, Taiwan.; 2 Whole-Genome Research Core Laboratory of Human Diseases, Chang Gung Memorial Hospital, Keelung, Taiwan.; 3 Department of Mathematical Sciences, Florida Atlantic University, Florida, US.; 4 School of Pharmacy, Institute of Clinical Pharmacy and Pharmaceutical Sciences, College of Medicine, National Cheng Kung University, Tainan, Taiwan.; 5 Department of Pharmacy, Keelung Chang Gung Memorial Hospital, Keelung, Taiwan.; 6 College of Medicine, Chang Gung University, Taoyuan, Taiwan.

**Keywords:** Chronic kidney disease, Low protein diet, Metabolites, Microbiota, Meta-analysis, Protein, Systematic review

## Abstract

**Background:** Although associations between low protein diet (LPD) and changes of gut microbiota have been reported; however, systematic discernment of the effects of LPD on diet-microbiome-host interaction in patients with chronic kidney disease (CKD) is lacking.

**Methods:** We searched PUBMED and EMBASE for articles published on changes of gut microbiota associated with implementation of LPD in CKD patients until July 2021. Independent researchers extracted data and assessed risks of bias. We conducted meta-analyses of combine p-value, mean differences and random effects for gut microbiota and related metabolites. Study heterogeneity was measured by Tau^2^ and I^2^ statistic. This study followed the Preferred Reporting Items for Systematic Reviews and Meta-Analyses guidelines.

**Results:** Five articles met inclusion criteria. The meta-analyses of gut microbiota exhibited enrichments of Lactobacillaceae (meta-*p*= 0.010), Bacteroidaceae (meta-*p*= 0.048) and Streptococcus anginosus (meta-*p*< 0.001), but revealed depletion of Bacteroides eggerthii (*p*=0.017) and Roseburia faecis (meta-*p*=0.019) in LPD patients compared to patients undergoing normal protein diet. The serum IS levels (mean difference: 0.68 ug/mL, 95% CI: -8.38-9.68, *p*= 0.89) and pCS levels (mean difference: -3.85 ug/mL, 95% CI: -15.49-7.78, *p* < 0.52) did not change between groups. We did not find significant differences on renal function associated with change of microbiota between groups (eGFR, mean difference: -7.21 mL/min/1.73 m^2^, 95% CI: -33.2-18.79, *p*= 0.59; blood urea nitrogen, mean difference: -6.8 mg/dL, 95% CI: -46.42-32.82, *p*= 0.74). Other clinical (sodium, potassium, phosphate, albumin, fasting sugar, uric acid, total cholesterol, triglycerides, C-reactive protein and hemoglobin) and anthropometric estimates (body mass index, systolic blood pressure and diastolic blood pressure) did not differ between the two groups.

**Conclusions:** This systematic review and meta-analysis suggested that the effects of LPD on the microbiota were observed predominantly at the families and species levels but minimal on microbial diversity or richness. In the absence of global compositional microbiota shifts, the species-level changes appear insufficient to alter metabolic or clinical outputs.

## Introduction

The global prevalence of Chronic Kidney Disease (CKD) is 9.1%; however, the burden of disease is increasing affecting 697.5 million people worldwide 1. The disease has significant impact on metabolic complications, cardiovascular disease, quality of life and mortality. Progression of CKD into end stage renal disease (ESRD) can lead to high medical financial demands. Understanding of pathophysiology of CKD and various management approaches are mandatory in reducing disease burden and its complications. Altered gut-renal interaction and gastrointestinal dysbiosis have been extensively described in CKD patients. The leak gut leads to bacterial translocation, causing micro-inflammation, abnormal immunity and production of noxious metabolites, and further aggravates the uremic toxicity. Significant intestinal bacterial overgrowth and changes of gut microbiota diversity and composition have been observed in CKD patients 2-4. Dietary counseling, including restriction of salt, potassium and phosphate intakes, represents important components in the care of renal patients. In particular, dietary protein restriction is commonly recommended in moderate to advanced CKD patients to reduce production of uremic wastes. Low protein diet (LPD), defined as daily intake < 0.8 g/Kg body weight, can decrease sodium loading, regulate sympathetic and angiotensin pathway, ameliorate urea and nitrogenous wastes and improve intraglomerular pressure resulting in reduced proteinuria and uremia 5-8. Clinical studies have indicated that the use of very low protein diet (VLPD, 0.4-0.6 g/Kg body weight/day) supplemented with ketoanalogues amino acids can further retard renal progression and reduce mortality 5, 9-11. Although associations between dietary protein restriction and preservation of renal function have been reported; however, the results remain ambiguous from diverse studies 12-14. As such, knowledge on the diet-microbiome-host interaction associated with implementation of LPD remains to be elucidated in CKD patients.

The diet provides substrate for intestinal fermentation and plays a role in modulating gut microbiota composition, altering production of diverse endogenous metabolites and determining disease progression 15, 16. Although an association between LPD and changes of gut microbiota has been indicated, it is unclear if changes of microbiota induced by dietary protein restriction can have beneficial effects on the outcomes of CKD patients. Diverse studies have indicated changes of gut microbiota associated with dietary interventions 17-21. Black et al. did not find changes of composition and diversity of gut microbiota 17; Lai et al have reported variations of relative abundances of gut microbiota at the family-levels 18; Jiang et al described changes at the genus-level 19; Di Lorio 20 and Wu et al. 21 revealed alterations in all three taxonomic levels, including bacterial families, genera and species, in patients undergoing LPD compared to those receiving normal diet. In addition to the modifications of microbiota in the gut of patients receiving LPD, the associated alterations of related surrogate indices of outcome, such as clinical, metabolomic and anthropometric parameters, have been described inconsistently in these studies. The interpretation of the results across these studies was limited by imbalanced baseline characteristics, small sample size, and differences in the analytic methodology and taxonomic classification reference. To our knowledge, systematically analysis and meta-analyses of the effects of LPD on gut microbiota in patients with CKD are still lacking. To fill this gap, we conducted a systematic review and meta-analysis which evaluate the roles of LPD in modulating gut dysbiosis in patients with CKD not yet on dialysis.

## Materials and Methods

### Design and search strategy

**Figure 1:**
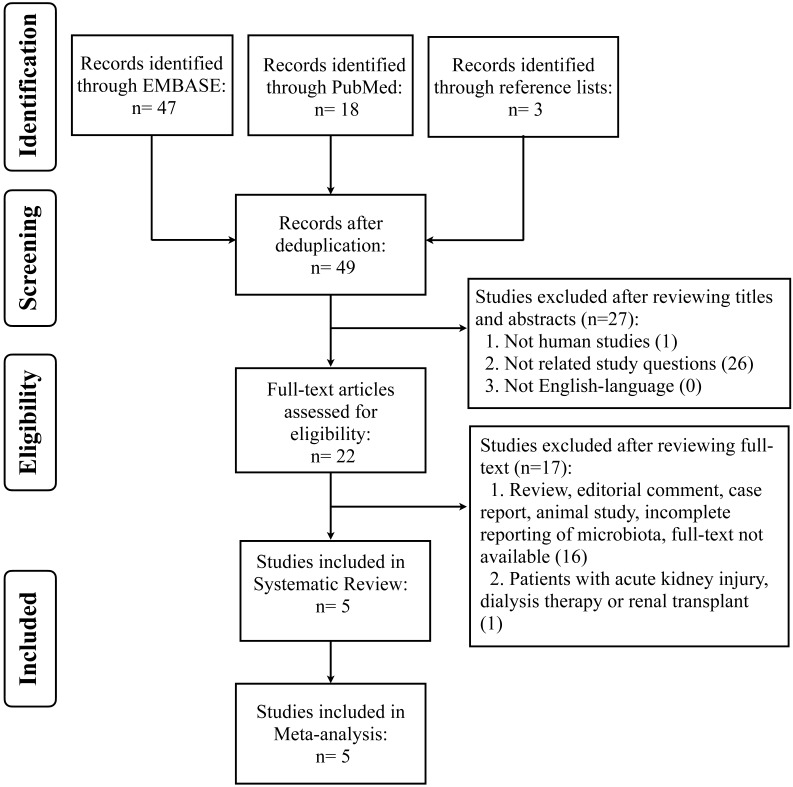
Preferred Reporting Items for Systematic Reviews and Meta-Analyses (PRISMA) flow chart on selection and inclusion of studies.

We conducted systematic review and meta-analysis of retrospective, cohort, case-controlled and randomized controlled studies in published literature from PUBMED and EMBASE, until July 17, 2021. The search strategy was based on the Population, Intervention, Comparator, Outcomes, Studies (PICOS) framework and involved the uses of synonyms and medical subject headings (MesH) or Emtree terms, including “renal insufficiency or CKD”, “protein restriction or LPD”, and “microbiota or microflora” (Table S1). Search terms were combined with Boolean operators (OR/AND). Studies were required to provide data on effects of LPD on microbiota (global bacterial composition or abundances of specific bacterial groups across different phylogenetic levels) in CKD patients. The reference lists of included articles were also hand-searched for relevant studies. Articles were managed using EndNote X9 (Analytics, Philadelphia, USA) to remove the duplicates. This study adhered to the reporting guidelines of Preferred Reporting Items for Systematic Reviews and Meta-analyses (PRISMA) 22 (Figure 1), and it has been recorded in the International Prospective Register of Systematic Reviews (PROSPERO) database (CRD42021238979).

### Literature selection

Two independent researchers (CYC and IWW) screened titles and abstracts to identify potentially eligible studies for full-text review. Original articles in English language were reviewed. Those studies involving patients with CKD not yet on dialysis, aged above 18 years old, undergoing LPD as an intervention and describing gut microbiota as outcomes of interest were included for analysis. Normal renal function controls were also included for comparative analysis (non-LPD group), depended on the original design of included studies. On the other hand, those studies enrolling patients aged below 18 years old, studies involving only animal models or reporting incomplete data of gut microbiota were excluded for analysis. Case report, reviews, consensus report, full-text not available and editorial article were also excluded. Studies involving patients with acute kidney injury, dialysis therapy or renal transplant were excluded.

### Data extraction and study quality

Data extraction was completed in duplicate by 2 independent reviewers (CKH, KJY). Information pertinent to research protocol, including site location, study design, year, definition, arms and duration of intervention were described. Participants' information regarding ethnicity, sample size, CKD stage, age and gender were extracted. Reporting of primary (microbiota characteristics: analytical methodology, database reference, bacterial diversity, taxonomic abundance) and secondary outcomes (biochemistry profiling or other measurements) were also conducted. For all included studies, mean ± standard deviation (SD) or median and interquartile range were used for data extracted. We contacted the study authors regarding possible incomplete data on the means and of selected outcome reporting. The studies have been excluded if a response was not received after three reminders and/or after attempting to contact another author of the study with no response.

Methodological quality of included studies was assessed independently by 2 authors (LCC, SCSu) based on the Risk of Bias in Non-Randomized Studies of Intervention (ROBINS-I) assessment tool 23. This tool includes seven specific bias domains, including: (1) assessment of confounding factors; (2) selection of participants; (3) classification of intervention; (4) deviation from interventions; (5) missing outcome data; (6) measurement of outcomes; and (7) selection of reported result. Risk of bias was rated as 1-low risk; 2-moderate risk; 3-serious risk; 4-critical risk; and 0-no information. When the reviewers' assessments differed with regard to data extractions or study quality evaluations, the additional reviewer (SCShao) were drawn in, on a case-by-case basis, to discuss and make the final judgments.

### Data synthesis and statistical analysis

We calculated the overall treatment effect for primary and secondary outcomes for each study included, using between-group differences as a measure of treatment effect. Data from cross-over interventions will be analyzed as pooled results (for study of Di Lorio et al. 20). Meta-analyses were undertaken for outcomes that were reported on by at least 2 studies. Effect sizes between-group differences in bacterial relative abundances and metabolite concentrations were calculated as the mean difference, standardized mean difference or 95% confidential interval (CI). The random effects model and mean differences were used for the meta-analysis. The overall effect of the intervention in relation to statistical significance was based on *p* < 0.05, and the results of the meta-analysis were presented as forest plots. We also applied meta-analysis methods for combining multiple expression profiles comparisons 24. For this method, we transformed two-sided *p* values collected from individual studies to one-sided p values for the consideration of heterogeneity among studies and combined p values analysis using Fisher's method as meta-analysis in microbiota analyses. Statistical heterogeneity among the studies was investigated by calculating Tau^2^ and the extent of heterogeneity attributable to heterogeneity was measured by the I^2^ statistic. Meta-analysis was performed using RevMan version 5.3.5 (Cochrane Collaboration, Oxford, UK). Statistical significance was defined as *p* < 0.05.

## Results

We identified 65 records, form PUBMED (n=18) and EMBASE (n=47) databases, and 3 hand-searched references for the initial assessment and only 22 articles were included for full-text review. Finally, five studies which met the inclusion criteria were selected for systematic review and meta-analysis (Figure 1).

Four of them were controlled trials 18-21 and one was prospective observational study 17. For those studies having more than one nutritional therapy 18, 20, only the LPD group rather than the VLPD or supplemented-LPD was selected as the intervention group for analysis. For the study of Di Lorio et al. 20, the Mediterranean diet, containing protein intake of 0.6-0.8 g/kg body weigh/day, was assigned as the LPD group. Overall, 239 patients (109 patients receiving LPD and 130 patients with normal-protein diet, NPD) were pooled for analysis. Although four of five articles have reported changes of gut microbiota in patients undergoing dietary intervention; however, the descriptions of microbial phylogenetic taxonomy classification were inconsistent, including families, phyla, genera and species-levels information 18-21. One article reported clustering analysis of gut microbiota rather than microbial taxonomic changes 17. The variations of indoxyl sulfate (IS) and p-cresyl sulfate (pCS) associated with changes of gut microbiota were described in three of all studies 17, 20, 21. The characteristics of the studies included are described in Table 1.

The assessments of risks of bias in the included studies are illustrated in Table 2. Three studies included in this review demonstrated serious, moderate and unknown risk of bias in terms of possible confounding effects from baseline and selection of participants 17, 19. Specifically, Black et al prospectively followed-up 30 pre-dialysis CKD patients who received LPD prescription and assigned the participants into adherent-LPD or non-adherent group according to their compliance to nutritional instructions rather than a pre-determined intervention assignment 17. Jiang et al. included 36 hospitalized CKD stage 5 patients having different dietary protein regimens 19; however, causes of inpatient care were not reported and may have affected the study outcome. Lai et al enrolled 16 CKD stage 3G-4G patients and 16 gender and renal function-matched controls to the study; however, the descriptions of baseline characteristics for the participants were lacking 18. The different domains of assessments of risks of bias of included studied are summarized in Table 2.

All studies applied 16S rRNA sequencing to decipher the composition of gut microbiota in patients undergoing LPD 18-21, except the study from Black et al. 17, where polymerase chain reaction and denaturing gradient gel electrophoresis for microbiota clustering analysis were used. The SILVA database catalogue was used in two studies to determine taxonomic classification of intestinal microbes. The effects of LPD on gut microbiota of patients receiving LPD are outlined in Table 3.

The diversities of gut microbiota community were assessed in four studies and no significant change on the α- and β-diversity among patients undergoing different dietary protein restriction was found, except for the study of Wu et al. 21. The changes in relative abundances of operational taxonomic units (OTUs) of two microbial families (Lactobacillaceae and Bacteroidaceae), one genus (Escherichia) and six species (Faecalibacterium prausnitzii, Coprococcus eutactus, Streptococcus anginosus, Bacteroides eggerthii, Blautia hydrogenotrophica and Roseburia faecis) were reported in more than two studies (Table S2). The meta-analyses of gut microbiota exhibited enrichments of Lactobacillaceae (meta-*p*= 0.010), Bacteroidaceae (meta-*p*= 0.048), Coprococcus eutactus (meta-*p*= 0.120) and Streptococcus anginosus (meta-*p*< 0.001) as well as revealed depletion of Bacteroides eggerthii (*p*=0.017), Blautia hydrogenotrophica (meta-*p*=0.225) and Roseburia faecis (meta-p=0.019) in patients receiving LPD compared to NPD patients. Although the mean effects of pooled studies have indicated an increase of mean relative abundances of Escherichia (meta-*p*= 0.444) and Faecalibacterium prausnitzii (meta-*p*= 0.340), the directions of changes of gut microbiota were inconsistent across the studies (Table 4).

**Figure 2:**
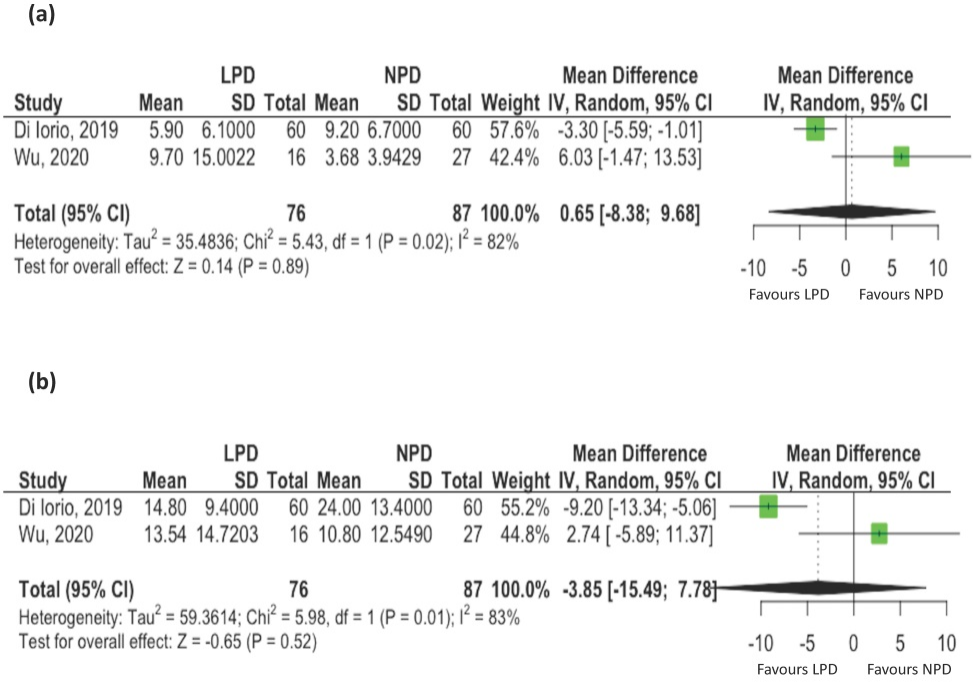
** The effects of low protein diet on uremic toxins associated with changes of gut microbiota. (A)** Indoxyl sulfate; **(B)** p-cresyl Sulfate (Black et al. conducted observational study and the mean values of uremic toxins were not available. Only these two controlled studies were pooled for metaanalysis).

Three studies reported changes of uremic toxins, including IS and pCS 17, 20, 21. Two studies reporting mean values were included for meta-analyses. The serum levels of total IS [mean difference: 0.68 ug/mL, 95% CI: -8.38-9.68, *p*= 0.89, Figure 2A) and total pCS (mean difference: -3.85 ug/mL, 95% CI: -15.49-7.78, *p* = 0.52, Figure 2B) did not change with LPD compared to NPD patients. However, there was evidence of statistical heterogeneity among the studies (I^2^ = 82%, *p*=0.02 for IS; I^2^ = 83%, *p*=0.01 for pCS).

**Figure 3:**
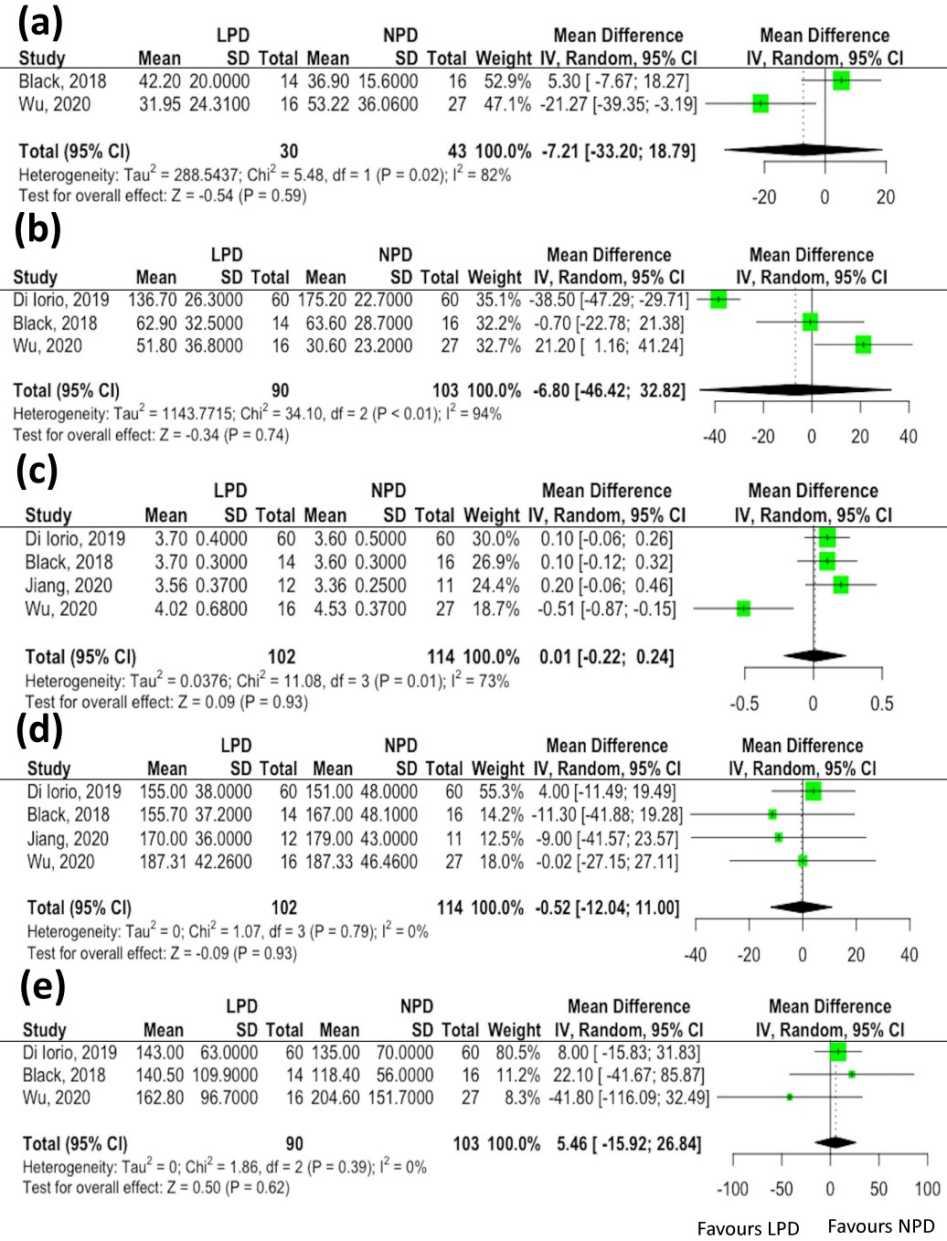
The effects of low protein diet on clinical parameters associated with changes of gut microbiota. **(A)** estimated glomerular filtration rate; **(B)** blood urea nitrogen; **(C)** serum albumin; **(D)** total cholesterol; **(E)** triglycerides.

Twelve serum clinical parameters (blood urea nitrogen, serum creatinine, sodium, potassium, phosphate, albumin, fasting sugar, uric acid, total cholesterol, triglycerides, C-reactive protein and hemoglobin levels) and three anthropometric estimates (body mass index, systolic blood pressure and diastolic blood pressure) associated with changes of gut microbiota were reported in more than two studies. We did not find significant differences in data indicative of renal function in association with changes of microbiota in patients receiving LPD compared to the NPD group [estimated glomerular filtration rate (eGFR), mean difference: -7.21 mL/min/1.73 m^2^, 95% CI: -33.2-18.79, *p*= 0.59, Figure 3A; blood urea nitrogen, mean difference: -6.8 mg/dL, 95% CI: -46.42-32.82, *p*= 0.74, Figure 3B]. Similarly, the serum albumin (mean difference: 0.01 mg/dL, 95% CI: -0.22-0.24, *p*= 0.93, Figure 3C), total cholesterol (mean difference: -0.52 mg/dL, 95% CI: -12.04-11.00, *p*= 0.93, Figure 3D) and triglycerides (mean difference: -15.92 mg/dL, 95% CI: -116.09-32.49, *p*= 0.62, Figure 3E) levels did not vary between the two groups. The meta-analyses of other clinical parameters (sodium, *p*=0.43; potassium, *p*=0.62; phosphate, p= 0.18; fasting sugar, *p*=0.39; uric acid, *p*=0.61; C-reactive protein, *p*=0.83 and hemoglobin, *p*=0.95) and three anthropometric estimates (body mass index, *p*=0.32; systolic blood pressure, *p*=0.31 and diastolic blood pressure, *p*=0.77) revealed not significant differences in these parameters associated with changes of gut microbiota between patients undergoing LPD vs. NPD.

Overall, significant changes of gut microbiota, predominantly at the families and species levels but minimal on microbial diversity or richness, were associated with use of LPD in CKD patients. In the absence of modification in the architecture of global microbiota community but presence of heterogeneity and bias of some studies, the changes of abundances of selected gut microbes appeared insufficient to shift metabolic or clinical output.

## Discussion

Comprehensive discernment of effects of dietary therapy on the intestinal dysbiosis remains incompletely elucidated in CKD patients. Protein restriction is the most frequent dietary intervention given to renal patients, in addition to the salt and water restriction. The results of this systematic review and meta-analysis have revealed significant changes of gut microbiota, mainly in the enrichments for *Lactobacillaceae*, *Bacteroidaceae* and S*treptococcus anginosus* and depletion of B*acteroides eggerthii* and *Roseburia faecis*, in patients receiving LPD compared to NPD group. In contrast, the microbiota changes were not associated with significant variations in gut-producing uremic toxins, renal function, and other clinical indices.

Although several studies have described dysbiosis of gut microbiota associated with LPD, the results are varied and were inconclusive, in terms of taxonomic classification, participants setting regarding to the disease severity and small sample size which limited statistical power. To fill this gap, the present work, using rigorous criteria on the assessments of eligibility of included studies, has recapitulated all literatures relevant to the changes of gut microbiota associated with LPD and has combined information across multiple studies to increase sensitivity. Common meta-analysis methods mainly combine three different types of statistics: combine *p*-values, combine effect sizes and combine ranks 25. Because of irregularity in reporting bacterial taxonomic abundances across studies, we applied a meta-analysis method combining *p* values using Fisher's statistics. This method has demonstrated satisfactory performance on the detection capability, biological association, model stability and robustness of study 24 warranting sufficient statistical power to the study.

Intestinal dysbiosis and compositional differences of gut microbiota have been described in renal patients 4, 26, 27. We did not find changes in microbiota diversity with dietary protein restriction. However, the LPD was associated with restoration of some but not all of the abundances of microbiota of CKD patients compared to health subjects. The abundances of butyrate-producing bacterial families, including* Lactobacillaceae*, *Bacteroidaceae* and *Prevotellaceae*, were lower in patients with uremia, whereas the population of indole- or p-cresol-producing bacterial families, such as *Enterobacteriaceae*, *Clostridiaceae*, and *Verrocomicrobiaceae*, were expanded compared to health individuals 4, 28, 29. While information on these tryptophanase possessing bacteria was incompletely unraveled from the included studies, this meta-analysis study found an increase in the abundances of *Lactobacillaceae* and *Bacteroidaceae* in patients receiving LPD. *Lactobacillus* species can metabolize tryptophan into indole-3-aldehyde, which increase the production of interleukin-22 through acting on the aryl hydrocarbon receptor in intestinal immune cells, ultimately controlling intestinal epithelial homeostasis 30, 31. The indole can further be metabolized by the liver into IS and became accumulated in CKD patients because of reduced renal excretion leading to renal progression 32. Administration of strains of *Lactobacillus salivarius* was associated with reduction of serum levels of both IS and pCS 33. The enrichment of *Lactobacillaceae* found in patients receiving LPD may in part explain the decreased serum levels of IS and pCS found in various included articles of this study 17, 20. *Faecalibacterium prausnitzii* is another proposed gut microbe associated with serum levels of IS or pCS; however, the abundance of this bacteria did not differ between LPD vs. NPD groups in our meta-analysis study. Two of three included studies 17, 20, 21 have shown reduction of uremic toxins after implementation of LPD in Caucasian population 17, 20. However, our findings did not suggest lowering of uremic toxins in association with dietary protein restriction. Furthermore, study heterogeneity, especially on stages of CKD, residual renal function, and discrepancy of dietary composition among participants across different countries, may have impacts on the actual association of serum levels of uremic toxins with variation of microbiota in CKD patients receiving LPD.

We found enrichment for intestinal *Bacteroidaceae* families but depletion of *Bacteroides eggerthii s*pecies in LPD patients. The later bacteria were found to increase in non-CKD subjects 4. These anaerobic bacteria produce enzymes responsible for breakdown of complex plant polysaccharides (such as cellulose and hemicellulose) and host-derived polysaccharides (such as mucopolysaccharides) generating phenolic acids 34. Main phenolic metabolites (phenylacetic acid, 4‐hydroxylphenylacetic acid and indole‐3‐acetic acid), can modulate mucosal glycosylation and promote angiogenesis and immune maturation. Yet, levels of these metabolites were only described in one included study 17. The relationships among changes of *Bacteroidaceae* abundance, related metabolites and renal outcome remain to be determined. *Streptococcus anginosus*, which increased in condition of unhealthy microbiome 35, was associated with tumorigenesis of intestinal tract, such as colon or esophageal cancer, and lupus activity 36-38. The abundance of this bacterium was lower in healthy subjects compared to CKD patients 4. The variation of this microbe observed in our study may confirm existence of intestinal dysbiosis of CKD patients. Finally, *Roseburia faecis*, a butyrate-producing anaerobic bacteria, can derive short-chain fatty acids (SCFAs) from fermentation of dietary fiber. Common SCFAs, including acetate, propionate and butyrate, may exert anti-inflammation, anti-atherosclerosis and anti-oxidative functions leading to amelioration of kidney damage 39, 40. The abundances of *Roseburia* were higher in healthy subjects than CKD patients 4. Only one included study reported associations of SCFAs with changes of gut microbiota in patients having LPD 21. The changes of abundances of *Bacteroidaceae* families, *Bacteroides eggerthii* and *Roseburia faecis* species found in this study may in part explain the variation of SFCA observed in patients receiving LPD 17, 21. The absence of tangible changes of abundance of these bacteria may in part explain the scanty impact on renal function associated with LPD. Although the inferences of effectiveness of nutritional intervention on the microbiome-metabolite axis were present in this meta-analysis; however, the subsequent impacts on the renal outcome of CKD patients should be validated in further large-scale studies.

Dietary components constitute important modulators of microbiota composition and function, which in turn affects the absorption, metabolism and storage of ingested nutrients, promotes intestinal barrier integrity, regulates mucosa inflammation and produces endogenous metabolites, resulting in intense effects on host physiology 41, 42. Although the adulthood microbiota is resilient, significant interindividual variability and plasticity of the gut microbiota have been observed. Changes in abundance or richness of certain gut microbial groups were identified in many chronic metabolic disease compared to healthy subjects 42. Microbiota remodeling creates an exceptional opportunity, by manipulating various external factors such as the diet or by transferring candidate gut microbiota, to reshape the architecture and biological functions of gut microbes for improved human health 15. Implementation of dietary intervention consisting of 12-week energy-restricted high-protein and weight-maintenance diet was able to elevate gut microbial gene richness and to alter enterotypes in obese and overweight individuals 43. The mechanisms by which changes in the dietary protein components and communities of gut microbiota regulate metabolic control are still evolving. The amino acids of diet provide gut microbes essential carbon and nitrogen for their metabolism function 15. Global serum metabolomics analysis has detected alterations of 130 metabolites in patients undergoing LPD compared with those receiving moderate-protein diet and has reported significant differences in the serum levels of 32 metabolites between participants assigned to the VLPD compared to those receiving LPD 44. Lobel et al. demonstrated that high sulfur amino acid-containing diet can not only induce posttranslational modification of microbial tryptophanase activity, leading to reduced cecal levels of IS, but also can mitigate kidney injury without altering microbial community composition in adenine induced-CKD mice 45. Gut microbial gene enrichments of D-alanine metabolism, synthesis/degradation of ketone bodies and glutathione metabolism were also observed in the CKD patients receiving protein restriction 21. Other possible mechanisms have been also documented, including altering circulating levels of uremic toxins 17, 20, tumor necrosis factor alpha (TNF-⍺), plasma nicotinamide adenine dinucleotide phosphate (NADPH) oxidase (NOX2) 18, SCFAs and bile acids 21 in association with changes of gut microbiota in patients receiving LPD. Further efforts to establish causal relationships of diet-microbiome interaction and to design prospects of personalized nutrition with specific dietary component manipulation should be necessary for CKD patients.

Although five studies were included in the overall systematic review and meta-analysis, merely two and three studies were included in the meta-analysis of gut microbiota and circulating markers, respectively. This could limit the generalizability of findings. Various possible reasons may contribute to the rarity of pertinent references on the research of gut microbiota in CKD patients undergoing LPD. First, good adherence to such a diet should need supports of multidisciplinary team (consisted of nephrologist, dietitian, case management nurse) to maximizing the compliance of patients. Second, research of microbiota requires sophisticated methodological collaboration of not only clinicians but also microbiologists, computing scientists and experts of bioinformatics and statistics to manage the megadata sets. Finally, the high cost of 16S rRNA sequencing may sometimes represent a barrier for large sample size studies. From all the included studies, Di Lorio et al. have conducted largest study enrolling 60 patients, by adopting cross-over design, to compensate the shortage in sample size of the study. We believed that the systematic review and meta-analysis may have an exceptional advantage to overcome the interpretation of individual studies having small sample size. Although the overall number of included studies remained low, the pooling of patients from all these researches may certainly increase the power and significance of studies. Therefore, more studies are needed in this area of research. In addition, differences in the reporting levels of gut bacterial abundances and in the taxonomic catalogue may hamper the comparison of actual effects of LPD across the studies included. Further high-throughput sequencing technology may facilitate in-depth studies of the gut microbiota associated with nutritional therapy. We used the “Mediterranean diet” as surrogate of LPD in the study of Di Lorio et al. 20; however, this diet is characterized by consumption of high contents of vegetables, fruits, legumes, nuts, beans, cereals, grains, fish and unsaturated fats, as well as low intake of meat and dairy foods. Extreme dietary protein restriction, namely the VLPD (< 0.4-0.6 g/ kg body weigh/day) often supplemented with ketoanalogues, may have additional effects on microbiota modulation 20, 46. We did not assess effects of VLPD on gut microbiota because of the scarce data presented in only two included studies. One inherent limitation to studying the health effects of particular diet is that some nutrients are rarely consumed in isolation. In consequence, experimentally handling of a specific nutrient can alter incorporation of other food components that may have metabolic effects unto themselves. For example, high-fat diets are commonly associated with LPD to avoid caloric malnutrition, which can also trigger effects on microbiota composition and metabolic consequences. Given the limitations of studying nutrients in isolation, a body of knowledge is accumulating to emphasize health effects of particular dietary pattern rather than experimental reductionism 41, 47. Lastly, in spite of the use of the random effects model in the analysis of clinical and metabolite markers, the high heterogeneity of included studies may have also affected the results of this meta-analysis. Thus, combination of different conventional meta-analysis methods may strengthen the robustness of models and the power of our study. We did not find variations of tangible renal outcome with LPD manipulation in CKD patients. Differences in patient selection, duration and composition of dietary intervention, and limited sample size may all contribute to the observed discrepancies with previous studies 5-8. Further prospective longitudinal studies with breakthrough methodologies, such as shotgun metagenomic sequencing, may help to elucidate the function of LPD intervention on mysterious intestinal microbiome-host metabolite synergies in order to preserve renal function of CKD patients.

## Conclusions

In conclusion, this systematic review and meta-analysis found that LPD can significantly alter the relative abundances of specific bacterial groups, including enrichments of *Lactobacillaceae*, *Bacteroidaceae* and *Streptococcus anginosus* as well as depletion of *Bacteroides eggerthii* and *Roseburia faecis*, in patients receiving LPD compared to NPD group. Our study suggested that the effects of LPD on the microbiota were observed predominantly at the families and species levels and relatively minimal on microbial diversity and richness. In the absence of global compositional microbiota shifts, the species-level changes appear insufficient to alter metabolic or clinical outputs. Furthermore, methodological aspects should be standardized to reduce potential bias and study heterogeneity and to allow interpretation of data in future studies. The findings of the present study, focusing on diet-microbiome-host interactions, provide insights for personalized diet recommendation potentially beneficial for CKD patients.

## Supplementary Material

Supplementary tables.

## Figures and Tables

**Table 1:** Summary of characteristics of included studies

First author	Year, country	Study design	Ethnicity	Total, n	CKD, n	CKD stage	Study arms, n	Intervention, n	Comparator, n	Definition of LPD	Age, mean	Male, n (%)	Primary outcome	Secondary outcome	Other measurements
Black, et al.	2018, Brazil	Prospective, observational	Latino-American	30	30	stage 3-4	2 (LPD adhesion vs. non-adhesion)	Adhesion group, 14	Non-Adhesion group, 16	≤0.6 g protein/kg/day for 6 months	55.8	16 (53.3%)	N/A (only reported clustering analysis)	Albumin, potassium, sodium, phosphate, urea, creatinine, uric acid, glucose, cholesterol, HDL-C, LDL-C, triglycerides, IS, PCS, IAA	Body fat, Lean mass, BMI, waist circumference
Di Lorio, et al.	2019, Italy	Prosective, randomized, crossover, controlled	Caucasian	60	60	stage 3B-4	3 (free diet, Mediterranean diet, VLPD)	Mediterranean diet group, 60	Free diet, 60	0.6-0.8 g protein/kg/day for 6 months	68.4	49 (81.7%)	Phylum, family, genus, species-levels	Albumin, creatinine, glucose, HbA1C, uric acid, sodium, potassium, calcium, phosphate, bicarbonates, cholesterol, triglycerides, ferritin, PTH, hemoglobin, CRP, urinary indices (sodium, potassium, phosphate, urea, total protein, creatinine), IS, PCS	BMI, systolic and diastolic pressure
Lai, et al.	2019, Italy	prospective and controlled	Caucasian	32	16	stage 3-4	3 (healthy vs. LPD vs. LPD + inulin)	LPD, 7	Health control, 16	≤0.6 g protein/kg/day for 6 months	N/A	N/A	Family-levels	Uric acid, CRP, IL-1β, IL-6, TNF-α, NADPH, NOX2	SF-36 Health Survey
Jiang, et al.	2020, China	Cross-sectional, controlled	Asian	40	36	Stage 5	3 (NPD vs. VLPD vs. LPD)	LPD, 12	NPD, 11	0.6-0.8 g protein/kg/day (duration: NA)	54	20 (50%)	Phylum, genus-levels	Albumin, creatinine, cholesterol, CRP	BMI
Wu, et al.	2020, Taiwan	Cross-sectional, controlled	Asian	77	43	Stage 2-5	3 (Healthy vs. LPD vs. NPD)	LPD,16	NPD,27	≤0.8 g protein/kg/day for 3 months	63.4	38 (49.4%)	Phylum, family, genus, species-levels	Albumin, urea, creatinine, hemoglobin, sodium, potassium, phosphate, glucose, uric acids, cholesterol, hs-CRP, urine protein-creatinine ratio, 11 saturated short-chain fatty acids, 41 bile acids, IS, PCS	BMI, systolic and diastolic pressure

Abbreviations: CKD, chronic kidney disease; LPD, low protein diet; VLPD; very-low protein diet; NPD, normal-protein diet; OTU, operational taxonomic units; HDL-C, high-density level cholesterol; LDL-C, low-density level cholesterol; IS, indoxyl sulfate; PCS, p-cresyl sulfate; IAA, indole acetic acid; HbA1C, glycosylated hemoglobin; PTH, parathyroid hormone; CRP, C-reactive protein; IL, interleukin; TNF, tumor necrosis factor alpha; NADPH plasma nicotinamide adenine dinucleotide phosphate; NOX2, NADPH oxidase; BMI, body mass index. N/A: not available.

**Table 2:** Risks of bias in included studies according to the Risk of Bias in Non-Randomized Studies of Interventions (ROBINS-I) tool

Author, year	Pre-intervention		At-intervention	Post-intervention				Overall risk of bias
	Domain 1	Domain 2	Domain 3	Domain 4	Domain 5	Domain 6	Domain 7	
	Bias due to confounding	Bias in selection of participants into study	Bias in classification of interventions	Bias due to deviation from interventions	Bias due to missing data	Bias in measurement of outcomes	Bias in selection of reported results	Unknown/low/moderate/serious/critical
Black et al., 2018	3	3	2	1	1	1	2	Serious
Di Lorio et al., 2019	1	1	1	1	1	1	1	Low
Lai et al., 2019	0	1	1	1	1	1	1	Unknown
Jiang et al., 2020	2	2	1	1	1	1	1	Moderate
Wu et al., 2020	1	1	1	1	1	1	1	Low

Risk of bias assessment: 0-No information; 1-Low; 2-Moderate; 3-Serious; 4-Critical.

**Table 3:** Summary of changes of gut microbiota and clinical parameters associated with low protein diet

First author	Microbiota methodology	Library catalogues	Changes of gut microbiota				Changes of biochemistry parameters*	Changes of other measurements*
			Diversity	Family-levels (RA of OTU)	Genera-levels (RA of OTU)	Species-levels (RA of OTU)		
Black, 2018	PCR, DGGE	N/A	⍺: no change; β: no change	N/A	N/A	N/A	**Decreased:** Cholesterol, LDL-C, PCS	No significant changes
Di Lorio, 2019	16S rRNA sequencing	Ribosomal Database Project, ver.10.28	⍺: no change; β: no change	**Increased:** Lachnospiraceae, Ruminococcaceae, Prevotellaceae, Bifidobacteriaceae **Decreased:** Lactobacillaceae, Streptococcaceae, Verrucomicrobiaceae, Enterobacteriaceae	**Increased:** Blautia, Bifidobacterium, Clostridium, Faecalibacterium, Coprococcus, Roseburia. **Decreased:** Ruminococcus, Collisella, Bacteroides, Lactobacillus, Akkermansia, Streptococcus, Escherichia	**Increased:** B. coccoides, B. hydrogenotrophica, B. obeum, B. wexlerae, B. adolescentis, B. pseudolongum, C. cadaveris, F. prausnitzii, C. eutactus, R. faecis **Decreased:** R. callidus, C. aerofaciens, B. uniformis, B. vulgatus, L. gasseri, L. salivarius, A. muciniphila, S. bovis, S. mutans, S. sobrinus, S. vestibularis, E. albertii	Increased: Sodium, bicarbonate, urine sodium. **Decreased:** Urea, phosphate, D-lactate, IS, PCS, urine phosphate	**Decreased:** Systolic and diastolic pressure
Lai, 2019	16S rRNA sequencing	SILVA database, ver. 132	⍺: N/A; β: no change	**Increased:** Akkermansiaceae, Bacteroidaceae. **Decreased:** Christensenellaceae, Clostridiaceae, Lactobacillaceae and Pasteurellaceae	N/A	N/A	**Increased:** Bicarbonate. **Decreased:** TNF-α, NOX2	**Increased:** Physical role function and general heath perception of SF-36
Jiang, 2020	16S rRNA sequencing	Genomes OnLine Database	⍺: N/A; β: N/A	N/A	**Increased:** Escherichia, Shigella **Decreased:** Blautia	N/A	N/A	No significant changes
Wu, 2020	16S rRNA sequencing	SILVA database, ver. 132	⍺: no change; β: increased	**Increased:** Ruminococcaceae. **Decreased:** Lachnospiraceae, Bacteroidaceae	**Increased:** Calditerricola, Coprococcus, Romboutsia, Parabacteroides, Alloprevotella, Subdoligranulum, Ruminococcaceae UCG-010, Faecalibacterium, Subdoligranulum, Cloacibacillus **Decreased:** Desulfovibrio, Pseudobutyrivibrio, Lachnospira, Eubacterium hallii group, Roseburia, Fusicatenibacter, Anaerostipes, Lachnoclostridium, Prevotellaceae NK3B31	**Increased:** Clostridium paraputrificum, Clostridium sordellii, Olsenella uli, Mogibacterium diversum, Blautia hydrogenotrophica, Lactobacillus mucosae, Porphyromonas gingivalis, Streptococcus anginosus, Lactobacillus sp. AB032. **Decreased:** Bacteroides coprophilus, Bacteroides plebeius, Bacteroides eggerthii	**Increased:** glyco λ-muricholic acid **Decreased:** eGFR, albumin, acetic, heptanoic and nonanoic acid	No significant changes

**Abbreviations:** LPD, low protein diet; OTU, operational taxonomic units; RA, relative abundances; PCR, polymerase chain reaction; DGGE, denaturing gradient gel electrophoresis LDL-C, low-density level cholesterol; IS, indoxyl sulfate; PCS, p-cresyl sulfate; TNF, tumor necrosis factor alpha; NOX2, nicotinamide adenine dinucleotide phosphate oxidase; eGFR, estimated glomerular filtration rate. N/A: not available. *Only those parameters showing significant differences between LPD vs. NPD (p<0.05) in the original articles were illustrated in this table.

**Table 4:** Meta-analysis of changes of microbiota in patients receiving low protein diet

Operational Taxonomic Units	*p* of study 1	*p* of study 2	Same direction	Mean effect	Fisher statistics	Meta *p*
*Families*						
Lactobacillaceae	0.0235	0.0615	Yes	LPD > NPD	13.07894592	0.010896423
Bacteroidaceae	0.013	0.639	Yes	LPD > NPD	9.581313492	0.048102986
*Genera*						
Escherichia	0.202	0.769	No	LPD > NPD	3.724303782	0.44460095
*Species*						
Faecalibacterium prausnitzii	0.8625	0.121	No	LPD > NPD	4.519769727	0.340209734
Coprococcus eutactus	0.2175	0.1185	Yes	LPD > NPD	7.316797494	0.120064405
Streptococcus anginosus	0.0175	0.00232	Yes	LPD > NPD	20.22348498	0.000451137
Bacteroides eggerthii	0.2065	0.012	Yes	LPD < NPD	12.00060699	0.017346752
Blautia hydrogenotrophica	0.0815	0.72	Yes	LPD < NPD	5.671312651	0.225076313
Roseburia faecis	0.0665	0.044	Yes	LPD < NPD	11.66823795	0.019996632

**Abbreviations:** LPD, low protein diet; NPD, normal-protein diet. “*p*” denoted *p* values.

## Abbreviations

CKD: chronic kidney disease
ESRD: end stage renal disease
LPD: low protein diet
VLPD: very low protein diet
PICOS: Population, Intervention, Comparator, Outcomes, Studies
MesH: medical subject headings
PRISMA: Preferred Reporting Items for Systematic Reviews and Meta-analyses
PROSPERO: International Prospective Register of Systematic Reviews
SD: standard deviation
ROBINS-I: Risk of Bias in Non-Randomized Studies of Intervention
CI: confidential intervals
NPD: normal protein diet
IS: indoxyl sulfate
pCS: p-Cresyl sulfate
OTUs: operational taxonomic units
SCFAs: short-chain fatty acids
