# Supplementary material for: Effects of Low Protein Diet on Modulating Gut Microbiota in Patients with Chronic Kidney Disease: A Systematic Review and Meta-analysis of International Studies
Source: Int J Med Sci. 2021 Oct 25;18(16):3839–50. doi: 10.7150/ijms.66451 (PMC8579282; doi:10.7150/ijms.66451)
Supplement: Supplementary file 1 — Supplementary tables. [file ijmsv18p3839s1.pdf]

**Supplementary Data**

## Effects of low protein diet on modulating gut microbiota in patients with chronic kidney disease: a systematic review and meta-analysis of international studies

Cheng-Kai Hsu[1#,] Shih-Chi Su[2#], Lun-Ching Chang[3], Shih-Chieh Shao[4,5], Kai-Jie Yang[1], Chun-Yu Chen[1], Yih-Ting Chen [1] and I-Wen Wu[1, 6]*

**Table S1:** Search terms and search strategy.

| **Patients/Population** | **Intervention** | **Outcome** | **Combined search terms** |
|---|---|---|---|
| Patients with CKD | Low protein diet | Gut microbiota | |
| **PUBMED** | | | |
| "renal insufficiency, chronic"[MeSH Terms] OR "chronic renal insufficiency"[All Fields] OR "chronic kidney disease"[All Fields] OR "CKD"[All Fields] | "diet, protein restricted"[MeSH Terms] OR "protein-restricted diet"[All Fields] OR "low-protein diet"[All Fields] | "microbiota"[MeSH Terms] OR "microbi-ota"[All Fields] | |
| **EMBASE** | | | Column 1 AND Column 2 AND Column 3 |
| chronic kidney failure'/exp OR 'chronic kidney disease' OR 'chronic kidney disorder' OR 'chronic kidney failure' OR 'chronic kidney insufficiency' OR 'chronic nephropathy' OR 'chronic renal disease' OR 'chronic renal failure' OR 'chronic renal insufficiency' OR 'kidney chronic failure' OR 'kidney disease, chronic' OR 'kidney failure, chronic' OR 'kidney function, chronic disease' OR 'renal insufficiency, chronic' | protein restriction'/exp OR 'diet, protein poor' OR 'diet, protein-restricted' OR 'low protein diet' OR 'protein free diet' OR 'pro-tein poor diet' OR 'protein restricted diet' OR 'protein restriction' OR 'pro-tein-restricted diet' | 'microflora'/exp OR 'microbiota' | |

Abbreviation: CKD, chronic kidney disease.

**Table S2:** Changes of gut microbiota associated with low protein diet in the included studies.

| Studies | LPD, n | LPD, mean | NPD, n | NPD, mean | p |
|---|---|---|---|---|---|
| ***Family*** | | | | | |
| Lactobacillaceae | | | | | |
| Di Iorio, 2019 | 60 | 0.127 | 60 | 0.052 | 0.047 |
| Wu, 2020 | 16 | 0.0524 | 27 | 0.0106 | 0.123 |
| Bacteroidaceae | | | | | |
| Di Iorio, 2019 | 60 | 0.023 | 60 | 0.0067 | 0.026 |
| Wu, 2020 | 16 | 0.0958 | 27 | 0.1054 | 0.722 |
| ***Genus*** | | | | | |
| Escherichia | | | | | |
| Jiang, 2020 | 12 | 0.0556 | 11 | 0.0204 | 0.404 |
| Wu, 2020 | 16 | 0.036 | 27 | 0.049 | 0.462 |
| ***Species*** | | | | | |
| Faecalibacterium prausnitzii | | | | | |
| Di Iorio, 2019 | 60 | 0.0219 | 60 | 0.025 | 0.275 |
| Wu, 2020 | 16 | 0.0591 | 27 | 0.0462 | 0.242 |
| Coprococcus eutactus | | | | | |
| Di Iorio, 2019 | 60 | 0.0016 | 60 | 0.0014 | 0.435 |
| Wu, 2020 | 16 | 0.0141 | 27 | 0.0112 | 0.237 |
| Streptococcus anginosus | | | | | |
| Di Iorio, 2019 | 60 | 0.0026 | 60 | 0.0005 | 0.035 |
| Wu, 2020 | 16 | 0.0043 | 27 | 0.0008 | 0.004 |
| Bacteroides eggerthii | | | | | |
| Di Iorio, 2019 | 60 | 0.001 | 60 | 0.0012 | 0.413 |
| Wu, 2020 | 16 | 0.0003 | 27 | 0.0023 | 0.024 |
| Blautia hydrogenotrophica | | | | | |
| Di Iorio, 2019 | 60 | 0 | 60 | 0.001 | 0.163 |
| Wu, 2020 | 16 | 0.001 | 27 | 0.0007 | 0.560 |
| Roseburia faecis | | | | | |
| Di Iorio, 2019 | 60 | 0.0038 | 60 | 0.0063 | 0.133 |
| Wu, 2020 | 16 | 0.0149 | 27 | 0.0206 | 0.088 |

Abbreviation: LPD, low protein diet; NPD, normal-protein diet.
